# An Integrated Framework for Data Quality Fusion in Embedded Sensor Systems

**DOI:** 10.3390/s23083798

**Published:** 2023-04-07

**Authors:** Christoph Scholl, Maximilian Spiegler, Klaus Ludwig, Bjoern M. Eskofier, Andreas Tobola, Dario Zanca

**Affiliations:** 1 Siemens AG, Technology, 91058 Erlangen, Germany; 2 Machine Learning and Data Analytics Lab, Department Artificial Intelligence in Biomedical Engineering (AIBE), Friedrich-Alexander-Universität Erlangen-Nürnberg (FAU), 91052 Erlangen, Germany; 3 Keyence Deutschland GmbH, 63263 Neu-Isenburg, Germany; 4 Institute of Electronics Engineering, Faculty of Engineering, Friedrich-Alexander-Universität Erlangen-Nürnberg (FAU), 91054 Erlangen, Germany; 5 Faculty of Electrical Engineering, Precision Engineering, Information Technology, Nuremberg Institute of Technology, 90489 Nürnberg, Germany

**Keywords:** data quality, domain knowledge, sensor systems, fuzzy logic, maximum likelihood, sensor fusion, embedded systems

## Abstract

The advancement of embedded sensor systems allowed the monitoring of complex processes based on connected devices. As more and more data are produced by these sensor systems, and as the data are used in increasingly vital areas of applications, it is of growing importance to also track the data quality of these systems. We propose a framework to fuse sensor data streams and associated data quality attributes into a single meaningful and interpretable value that represents the current underlying data quality. Based on the definition of data quality attributes and metrics to determine real-valued figures representing the quality of the attributes, the fusion algorithms are engineered. Methods based on maximum likelihood estimation (MLE) and fuzzy logic are used to perform data quality fusion by utilizing domain knowledge and sensor measurements. Two data sets are used to verify the proposed fusion framework. First, the methods are applied to a proprietary data set targeting sample rate inaccuracies of a micro-electro-mechanical system (MEMS) accelerometer and second, to the publicly available Intel Lab Data set. The algorithms are verified against their expected behavior based on data exploration and correlation analysis. We prove that both fusion approaches are capable of detecting data quality issues and providing an interpretable data quality indicator.

## 1. Introduction

Embedded sensor systems generate massive amounts of data for many different use cases. Applications include predictive maintenance, robotics, and health care. Many of these use cases have significant business impacts but are also often related to the safety or security of the monitored facility. It is crucial for these sensor systems to monitor their data quality level, ensuring the reliability of the observed process and the actual application. Embedded sensor systems are resource constrained, making it necessary to define and aggregate data quality-related attributes into a single meaningful indicator. This step is often necessary because of a limited communication bandwidth or power consumption budget. These constraints limit the ability of embedded sensor systems to transmit raw data frequently, making it necessary to transfer already preprocessed data.

**Figure 1:**
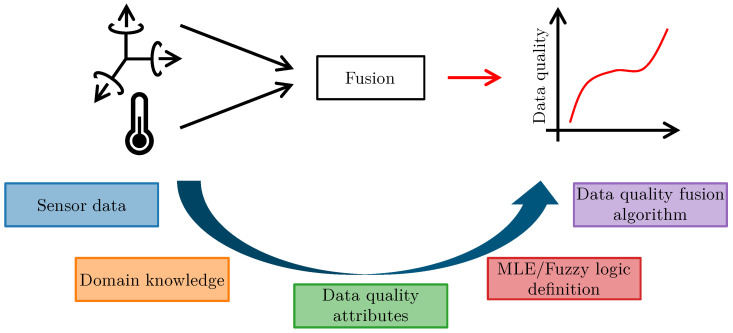
Overview of the proposed fusion framework to perform data quality indication.

Most sensor systems consist of a microcontroller, and one or several sensors embedded in one device [1]. These sensor systems communicate with their environment through classical fieldbuses, Ethernet networks, or wireless communication systems, such as Bluetooth or wireless local area networks (WLAN) [2]. They often face constraints such as communication bandwidth or battery life, limiting the ability to measure or transmit data at high frequencies [3]. Despite these constraints, sensor systems often monitor processes and events in areas where false data could lead to dangerous decisions. Areas of application include medicine [3], robotics [4], and infrastructure [5]. Thus, we emphasize the importance of enhancing sensor systems with capabilities to monitor their current level of data quality given a set of measurements. This work presents a framework for the knowledge- and data-driven fusion of data quality attributes of sensor systems in general and MEMS-based systems in particular. We present a holistic approach that covers the definition of data quality attributes and fusion algorithms, generating a single indicator that describes the overall quality of the data. The framework allows for the use of domain and expert knowledge to define fusion algorithms for data quality indication. The algorithms compute interpretable indicators with high information content, where higher values indicate higher overall data quality. Figure 1 gives an overview of the proposed framework.

The workflow consists of five steps. The goal is to transform real valued sensor measurements into data quality attributes and fuse them into a single meaningful and interpretable data quality indicator. The first and second steps utilize domain knowledge to derive data quality attributes based on the available sensor data. Then the fusion algorithms are defined based on statistical properties and expert knowledge. Finally, the process results in a data quality fusion algorithm, which outputs the calculated data quality indicator. The output of the fusion algorithm is a single meaningful number that represents the data quality of the observed sensor system. The proposed method is applied to two data sets. The first data set covers the temperature dependence of the sampling rate from an industry-grade MEMS accelerometer. The precision of the sampling rate of these systems influences the overall data quality, as algorithms such as frequency domain-based approaches require accurate sampling rates. The second data set is publicly available. It consists of distributed sensor systems that monitor environmental parameters such as temperature, light, and device-related metadata.

We bridge existing gaps by allowing the direct use of the data quality attributes, their metrics, and the defined bounds for data fusion, using maximum likelihood estimation and fuzzy logic. Both algorithms can fuse multiple inputs into one single meaningful indicator. The algorithms do not require scaling or normalization. All sensor readings can be input directly into the algorithms, addressing limitations of previous works in the field. In addition, we contribute a semi-automated approach to defining fuzzy logic membership and likelihood functions, making it possible to create quality fusion algorithms with minimal effort. Existing work requires manual specification of the properties of the fusion algorithms. Our semi-automatic approach limits the required amount of manual work to specify the properties of the algorithms. The predefined set of supported rules and distributions limits the initial degrees of freedom, supporting the quick setup of a data quality fusion algorithm and its test.

## 2. Related Work

Various approaches have been proposed to track the data quality of sensors. In [4], a capacitive sensor system in combination with an unsupervised learning algorithm is introduced. Their approach is to detect out-of-distribution samples, which is used to track data quality issues. D’Aniello et al. proposed to evaluate sensor data quality by estimating missing values with association rules [6]. This work is based on the definition of virtual sensors that meet service level agreements (SLA). These SLAs incorporate the required data quality level. Ehikioya et al. [7] have published another article on information quality focusing on business and enterprise data. They postulate that information quality is an engineered product created by an information manufacturing process and could be solved with fuzzy sets. The aspect of data quality from a metrology perspective has been addressed in [8,9]. Eichstädt et al. focused on tractability aspects with an emphasis on sensor calibration in the Internet of Things (IoT). Vedurmudi et al. introduced an ontology to describe aspects of data quality such as indicators, metrics, and interpretations semantically. An approach to fuse the characteristics of observed events, operational characteristics, and quality of information attributes based on statistical tests has been made in [10]. Data aggregation and fusion methods are widely used in sensor systems in the context of data quality indication and can be roughly clustered in probabilistic, statistical, and knowledge-based methods [11]. Probabilistic approaches include Kalman filter-based methods. Feng et al. proposed the combination of a robust Kalman filter and partial least squares regression for the simultaneous detection and correction of erroneous signals [12]. Sallans et al. have introduced a method to automatically detect abnormal sensor values based on statistical models [13]. Their method allows for the online update of model parameters, enabling the model to consider sensor drift and changing conditions. Knowledge-based approaches include fuzzy logic systems and have been introduced to solve data quality-related problems [6,7]. Wu et al. [14] proposed to normalize the range of individual metrics in the interval of [0,1] and fuse it by taking the product of all metrics.

Different authors have previously defined a variety of data quality attributes. These attributes reflect important aspects of sensor signals, influencing the signal quality of the complete system. A summary of commonly used attributes is given in Table 1. Data quality attributes can also be considered data quality dimensions because they represent different quality aspects of a signal. In a typical scenario, an embedded sensor system transmits preprocessed data, which are calculated from raw sensor data readings. Therefore, it is important to have a meaningful data quality indicator associated with a processed data sample ready for transmission. Limiting factors such as computational requirements, communication bandwidth, and energy budget should be considered. The fusion of quality attributes is a convenient approach to creating an indicator that contains as much information as possible. A widely used data set to test data quality indication methods is the “Intel Lab Data” data set [15].

Frequency domain-based methods have been used to monitor machine conditions [19]. A processing pipeline based on variable mode decomposition, total variation denoising, and second-order cyclostationary blind convolution has been proposed by Yang et al. [20]. Their method allows for the extraction of fault features from noisy signals in applications such as bearing diagnosis based on acceleration data. In the IoT context, MEMS sensors are widely used to acquire acceleration data [1] to perform frequency domain diagnosis. The temperature dependence of MEMS accelerometers has been studied by Martinez et al. [21], who proposed a compensation method to account for temperature bias in sensor output magnitude. Compensation is useful to prevent unwanted effects from sensors, such as bias or drift. However, in the simplest case, compensation does not acknowledge the fact that measurements can be wrong due to internal or external events.

## 3. Materials and Methods

This section introduces the methods and data used in this work. First, we introduce the overall architecture of the framework. Second, the used data quality attributes are defined, and the metrics used to calculate the indicators are established. Then, the fusion and aggregation algorithms are introduced. Finally, the data sets used for this work are described.

### 3.1. Data Quality Fusion Architecture

**Figure 2:**
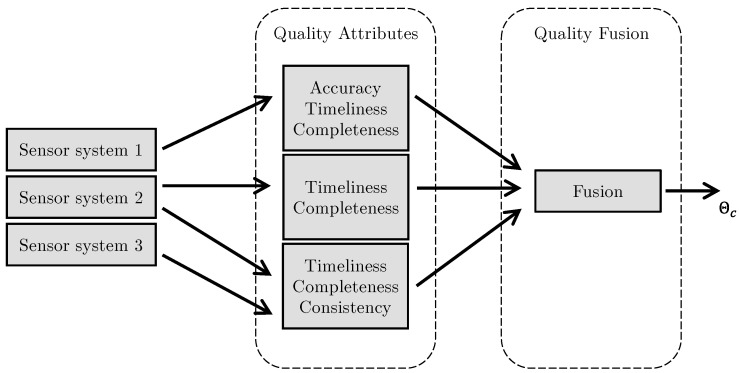
Data quality fusion architecture. Based on data from sensor systems, data quality attributes are calculated. Some attributes require input from multiple sensors. The attributes are passed into the fusion algorithm which outputs $\theta_{c}$, the combined data quality indicator.

Figure 2 shows the overall architecture to aggregate data quality attributes. Several sensor systems produce measured values, and data quality attributes such as timeliness, or completeness are defined. Each measured data point is associated with data quality attributes. Depending on the nature of the attribute, inputs from one or multiple sensors are required. Determining data quality attributes relies on available knowledge sources, such as plant operators, workers, or developers knowing a product. Additional sources of information are data sheets, as well as previously acquired data.

In order to aggregate multiple attributes derived from the metrics into a single data quality indicator, a data fusion algorithm is added to the architecture. The task of the fusion algorithm is to calculate a meaningful and interpretable value, representing the overall quality of the data in the complete system. Depending on the application scenario, a layered fusion approach is applied, resulting in a fused data quality indicator $\Theta_{1}\ldots \Theta_{N}$. These indicators can be used individually or combined once more into a combined indicator $\Theta_{C}$ that describes the quality of data from a set of sensors. The final quality indicator is outputted in the range of [0,1]. Interpretability is gained in the sense that lower values indicate lower data quality, whereas higher values indicate higher data quality.

### 3.2. Data Quality Attributes

This section introduces the attributes used for this work and defines the metrics for their calculation.

1. **Accuracy**
  This attribute is related to the error of a sensor signal with respect to a reference value or overall precision [17]. Usually, embedded sensor systems are not equipped with a calibrated sensor to reference their measured value. Accuracy can be related to sensor precision, which is information provided by the manufacturer. Furthermore, domain knowledge or data are sources to define accuracy metrics.
  Another approach called dynamic uncertainty, proposed by Gatian et al. [22] relates the sensor’s accuracy to uncertainty in the sense of rapid transient changes. The authors propose to use a sliding window to compute the standard deviation in a given time series and to compare it with a predefined threshold.
2. **Consistency**
  Inconsistent measurements deviate significantly from similar measurements, closely related in time and space. This attribute is related to the coherence between sensor signals and is calculated based on sensors that measure the same physical quantity. The consistency metric is defined by calculating the absolute error over two values as follows:
  (1) $$Consistency=|x_{1}\left(t\right)-x_{2}\left(t\right)|,$$
  where $x_{1}\left(t\right)$ and $x_{2}\left(t\right)$ are measurements taken at time point *t*. Both values represent measurements of the same physical quantity.
3. **Completeness**
  This quality parameter relates to missing values [18] in a series of measurements. In sensory applications, usually the acquisition of a certain number of samples is required to perform calculations such as frequency domain analyses. If a sufficient number of values are not acquired, this series is incomplete, calculated by the following metric:
  (2) Completeness=|X|.
  where |X| is a set of values acquired in a given time period. The cardinality of *X* represents the number of measured samples, giving a measure of completeness.
4. **Timeliness**
  The timeliness attribute is addressed in multiple use cases and is defined slightly differently depending on the usage. It is often related to its currency or up-to-dateness, an additional aspect in embedded sensor systems. Gutierrez et al. [17] define timeliness as the delay between the time point of data availability and the time point of data availability advertisement. According to the definitions in [18,23], we define timeliness in the sense of currency, which means how quickly data are updated. This definition allows for the application of timeliness to sensor systems, where a common task is to measure a quantity periodically. If the periodic interval is not maintained, this indicates a low data quality for the task at hand. The definition of timeliness is the difference between two consecutive timestamps of sensor readings:
  (3) $$Timeliness=t_{i}-t_{i-1}.$$
  The value $t_{i}$ is the current timestamp of a given sample, and $t_{i-1}$ is the timestamp of the previous sample.

### 3.3. Maximum Likelihood Estimation

Based on empirical data, statistical properties of the data quality attributes can be derived. Statistical properties are used to derive probability distributions $f_{i}$, given observations $x_{i}$ and the likelihood parameter θ:

(4) $$L\left(\theta \right)=\prod_{i=1}^{n}f_{i}\left(x_{i}|\theta \right).$$

The combined likelihood function *L* is the product of all individual probability functions *f* [24]. For independently and identically distributed observations, *f* is constant. If this is not the case, it is necessary to derive individual distributions for every value $x_{i}$. Here, observations represent a concrete realization of a data quality attribute and its associated metric. New observations are bound to the distributions; then the maximum likelihood parameter is obtained by maximizing *L* with respect to θ as follows [24]:

(5) $${\hat{\theta }}_{ML}=arg\;\underset{max}{\theta \in \Theta }\;L\left(\theta \right).$$

where Θ is the parameter space of θ. The maximized likelihood ${\hat{\theta }}_{ML}$ represents the probability of high or low quality of the data, given a set of observations. The parameter θ is part of the individual probability distributions. Distributions $f_{i}$ are parameterized so that observations in the sense of high data quality are closer to the maximum of a given distribution. Therefore, it is beneficial to bind θ to the parameter that controls the variance. The population mean μ and the standard deviation σ describe a normal distribution as follows [25]:

(6) $$f_{Normal}\left(x\right)=\frac{1}{\sigma \sqrt{2\pi }}e^{-{\left(\frac{x-\mu }{2\sigma }\right)}^{2}}.$$

The variance Var of a normal distribution is $Var=\sigma^{2}$. The likelihood parameter θ is embedded in the distribution function as follows:

(7) $$\sigma =\sigma_{\theta =1}\;\cdot \;\frac{1}{\theta }.$$

The value $\sigma_{\theta =1}$ represents the standard deviation, where the data quality parameter is in the high range. For an exponentially distributed data quality attribute, the steps are similar. The probability distribution $f_{Exp}$ is parameterized by the scale parameter β and the location parameter μ [25]:

(8) $$f_{Exp}\left(x\right)=\frac{1}{\beta }\;\cdot \;e^{-\frac{\left(x-\mu \right)}{\beta }}.$$

Simplifying α=1/β, the variance of an exponentially distributed value is $Var=1/\alpha^{2}$. The likelihood parameter θ is embedded in the exponential distribution as follows:

(9) $$\alpha =\alpha_{\theta =1}\;\cdot \;\theta .$$

**Figure 3:**
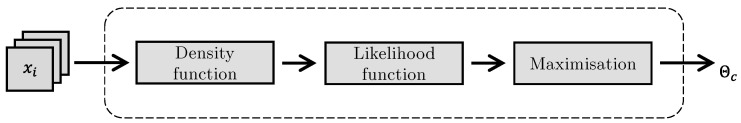
Maximum likelihood estimation based data quality fusion. Consisting of the steps probability density function, likelihood function and maximization.

Creating the combined data quality indicator based on a new set of observations with the maximum likelihood estimation method consists of three steps, as presented in Figure 3. First, observations $x_{i}$ are plugged into their respective probability distributions. The individual likelihood functions, parameterized by θ, are then joined into a single combined likelihood function that represents the current quality of the data. Finally, the combined likelihood function is maximized in the interval [0,1] to obtain the likelihood of high or low data quality, given the current observations.

### 3.4. Fuzzy Logic

Fuzzy logic extends the discrete two-valued binary logic to the continuous interval [0,1] [26]. A fuzzy set μ is the projection of the reference set *X* into the unit interval [0,1]:

(10) μ:X→[0,1].

The function μ is also called the membership function, which represents the degree of membership of an observation to a set. Frequently used membership functions include S-shaped, Z-shaped, and Gaussian membership functions (MF). The S-shaped MF is defined as follows:

(11) $$f\left(x;a,b\right)=\left\{\begin{array}{cc} 0 & x\;\leq \;a\\ 2{\left(\frac{x-a}{b-a}\right)}^{2} & a\;\leq \;x\;\leq \;\frac{a+b}{2}\\ 1-2{\left(\frac{x-b}{b-a}\right)}^{2} & \frac{a+b}{2}\;\leq \;x\;\leq \;b\\ 1 & x\;\geq \;b. \end{array}\right.$$

This membership function has two parameters, *a* and *b*. They control the points where the membership starts to be above zero and where the function reaches a membership of one. The parameter x represents the input, which is to be fuzzified. The Z-shaped membership function is defined similarly to the S-shaped type; however, the definition is in reverse order:

(12) $$f\left(x;c,d\right)=\left\{\begin{array}{cc} 1 & x\;\leq \;c\\ 1-2{\left(\frac{x-c}{d-c}\right)}^{2} & c\;\leq \;x\;\leq \;\frac{c+d}{2}\\ 2{\left(\frac{x-d}{d-c}\right)}^{2} & \frac{c+d}{2}\;\leq \;x\;\leq \;d\\ 0 & x\;\geq \;d. \end{array}\right.$$

The parameters *c* and *d* represent the points where the degree of membership starts to decay from one and where the function reaches zero. The Gaussian membership function is defined as follows:

(13) $$f\left(x;\sigma ,\mu \right)=e^{-{\left(\frac{x-\mu }{2\sigma }\right)}^{2}}.$$

**Figure 4:**
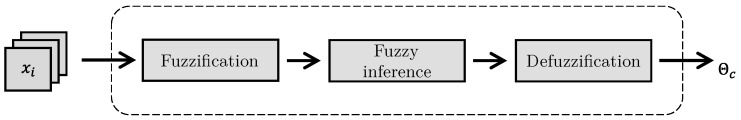
Fuzzy logic based data quality fusion. Consisting of the steps fuzzification, fuzzy inference and defuzzification.

Here, σ is the standard deviation and μ the mean of the input value. The concept of fuzzy sets is used to fuse data quality attributes based on domain knowledge and existing measurements. Figure 4 describes the general workflow of a fuzzy logic-based data fusion.

The membership functions are defined based on the inputs $x_{i}$, representing the data quality attributes. They transform the crisp input values into the fuzzy domain. Following this step, the fuzzy inference system defines the behavior of the fusion algorithm. To assign membership functions to ranges such as high data quality, low data quality, or intermediate values medium or average, domain knowledge is used. The bounds between the ranges are subject to design decisions taken when engineering the data quality indication algorithms. Next, in the fuzzy inference step, the logical relations between the fuzzified attributes and the overall level of data quality are defined. This step is again subject to design decisions. However, it is essential to cover all permutations of defined membership functions. If this is not the case, those memberships not covered in the inference step can be omitted, because they do not add information to the final output. For a system consisting of two data quality attributes with the assigned levels high and low, a fuzzy inference system could be designed as follows:

(14) $$R_{1}:Data\;quality\left[high\right]=Data\;quality\;attr.\;1[high]\;\wedge \;Data\;quality\;attr.2[high],$$

(15) $$R_{2}:Data\;quality\left[low\right]=Data\;quality\;attr.\;1[low]\;\vee \;Data\;quality\;attr.\;2[low].$$

This ruleset will only output a high data quality if both attributes are also high, using a fuzzy-and connection of the signals. Equation (15) demonstrates the case of low data quality. The attributes are or-ed, meaning that any low data quality sample results in low data quality

Defuzzification is the final step of the process. The results of the inference step are transformed back into a crisp output value, representing the current data quality θ. This step requires the definition of an output membership function, transforming fuzzy attributes such as low or high data quality into crisp output values.

### 3.5. Semi-Automatic Definition of Fusion Algorithms

To reduce manual labor during the initial setup of a data quality fusion algorithm, we propose a semi-automatic framework for the definition of likelihood functions or fuzzy logic membership functions. The data-driven approach uses available measurements or domain knowledge, such as data sheets, to create a working fusion algorithm with minimal effort. To enable the semi-automatic rule definition, a set of supported distributions is defined, such as normal or exponential. The measured observations or domain knowledge is fed into an array such that an observation resolves to an assigned data quality. For purely data-driven parameters, the histogram can be used as a basis, considering more frequent observations to have higher data quality compared to observations occurring less often. If domain knowledge is used to perform data quality indication, a table must be provided that maps observations to data quality levels.

**Figure 5:**
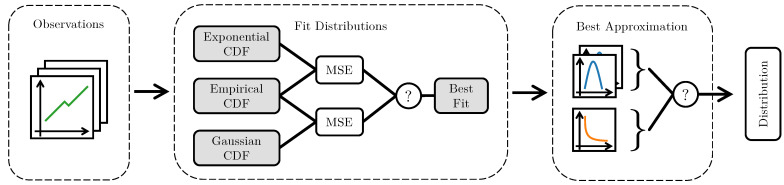
Semi automatic rule definition based on statistical properties of observations and majority voting.

The procedure for the semi-automatic definition of fusion algorithms is shown in Figure 5. The process works as follows: First, the data containing all observations of data quality attributes or mappings of data quality levels are fitted to the supported probability distributions. The empirical cumulative distribution (CDF) is then calculated, and the goodness-of-fit is evaluated concerning the fitted distribution. The evaluation metric for the goodness of fit is based on the mean squared error (MSE) between the empirical and fitted CDF. A lower MSE indicates a better quality of the fit, and thus a superior approximation of the underlying data. If multiple measurement series are available for the same data quality attribute, this process is repeated for all series. A majority vote allows the best fit for all series to be found, counting the number of fits per supported model. The model that is counted the most is considered the best. If multiple models are available, the model parameters, such as mean and standard deviation, can be averaged to find the most representative solution. In the example in Figure 5, three sets of observations are evaluated. The step “Best Approximation” chooses the Gaussian distribution because it was found to fit the data better in two cases, whereas the exponential case was better only once.

After finding the most suitable statistical model for the quality attributes, this information is further used to define quality fusion algorithms. The statistical model found in the previous step can be used directly for data fusion using maximum likelihood. For fuzzy logic-based fusion, bounds for high and low data quality need to be defined, which are derived from the statistical properties. This is achieved by defining multiples of the allowed variance of the fitted distributions centered around the found mean, which still represents a high data quality. A sufficient granularity of fuzzy representations is achieved by defining three membership functions, low, medium, and high. Low and high memberships represent poor data quality, as they are too far off compared to the expected mean and variance. The membership medium represents high data quality, as the newly captured observation is well within the expected range.

**Figure 6:**
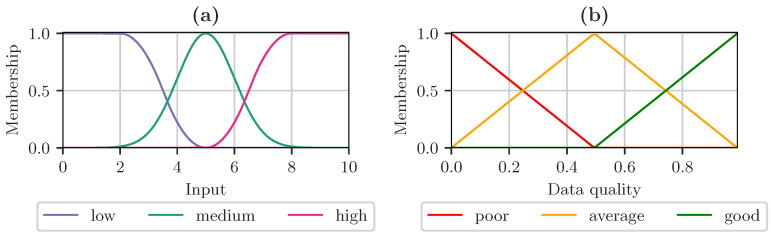
Example membership function for (**a**) an input between zero and ten. Z-shaped membership for low, Gaussian membership for medium, and S-shaped membership for high. (**b**) data quality with trapezoidal membership for poor, average and good.

The semi-automatic definition of fuzzy logic membership functions also relies on properties derived from data and observations. The support points of the membership functions are derived from the mean and standard deviation. The proposed framework supports Gaussian membership functions for average, Z-shaped membership functions for low, and S-shaped membership functions for high. An example of defined membership functions is given in Figure 6. The input range is defined between zero and ten. The mean of the Gaussian membership function is set to be five, and its standard deviation to one. The fuzzy logic algorithms were implemented in Python using the Scikit-Fuzzy library [27]. All implemented fuzzy systems are of type-1 Mamdani. The defuzzification method is bisector, the conjunction method is implemented as the minimum, and the disjunction method as the maximum.

The S- and Z-shaped membership functions are defined so that the points a, b, c, and d according to Equations (11) and (12) cross the maximum and minimum of each side of the Gaussian membership function, respectively.

### 3.6. Accelerometer Data Set

This data set was acquired based on a custom-designed PCB, equipped with an STMicroelectronics STM32L4A6 microcontroller. The sensor system carries an STMicroelectronics LSM6DSL inertial measurement unit, a Texas Instruments TMP461 temperature sensor, and a BOSCH BMP280 pressure and temperature sensor. The purpose of this data set is to measure the influence of temperature changes on the sample rate of LSM6DSL. This temperature dependence is used to derive data quality attributes such as accuracy, timeliness, completeness, and consistency based on the measured data and the available data sheet information. The sensors are configured as follows: LSM6DSL is configured to measure acceleration only, in FIFO mode (first in, first out buffer), at a frequency of 6664 Hz and a sensitivity of 2 mg [28]. Using the FIFO, the measured acceleration values of all three axes are buffered in the internal memory and retrieved asynchronously. The application is engineered so that the FIFO buffer is cleared well before it overruns. The data extraction period from the buffer is 50 ms. Configuration of the BMP280 temperature sensor is set to the oversampling factor 16, normal operation mode, and 125 ms sampling interval. Data are collected through the inter-integrated circuit bus (I2C) every 1000 ms. The pressure value measured by this device is not acquired. The settings applied to the temperature sensor TMP461 are left to default, according to the data sheet [29]. A new sample is acquired every 250 ms through the I2C bus. The timing and scheduling of the application are maintained using the real-time operating system FreeRTOS. All acquired data points are streamed out of the device using a proprietary UART (universal asynchronous receiver transmitter) protocol. A logging tool written in Python runs on a PC and logs the received data in files for further analysis. All data points are time-stamped based on a precision hardware timer running at 8.192 kHz. The sampling rate of the LSM6DSL sensor is measured based on the predefined interval of 50 ms, using the number of samples present in the FIFO buffer and the readout period. A total of three measured parameters are relevant for the fusion of data quality; these are the sample rate LSM6DSL, the temperature TMP461, and the temperature BMP280. To acquire the data set, three PCBs were placed in a VOETSCH VT 7010-type climate cabinet. The sensor systems were not attached to a moving object during these tests. The temperature in the cabinet was cycled repeatedly from 0 °C to 40 °C with a step size of 10 °C. Table 2 shows the properties of the data set. A total number of 3.5 million samples were recorded from every sensor system. In addition to the three sensor systems, a reference temperature sensor of type RS PRO RS-172TK was placed in the climate cabinet to track the temperature. Gaps in the data are filled by merging the closest value to the timestamps of the measured values. All data quality attributes introduced in Section 3.2 were applied to the data points in this data set. The accuracy attribute was mapped to the sample rate of LSM6DSL. The temperature dependency of the sampling rate directly influences the accuracy of this value with respect to the assumed value specified by the data sheet. A calibration was not performed regarding the LSM6DSL accuracy attribute. The setup uses the microcontroller and its timer module to ensure proper timing during data acquisition. Consistency was calculated between the temperature measured by TMP461 and BMP280. The completeness and timeliness were calculated for all three measured parameters.

The fuzzy logic rule set used for this data set is defined as follows:

(16) $$\begin{array}{cc} R_{1}:Data\;quality\left[poor\right]= & consistency_{temperature}\left[low\right]\;\vee \;completeness_{LSM6DSL}\left[low\right]\;\vee \\ & completeness_{TMP461}\left[low\right]\;\vee \;completeness_{BMP280}\left[low\right]\;\vee \\ & timeliness_{LSM6DSL}\left[low\right]\;\vee \;timeliness_{TMP461}\left[low\right]\;\vee \\ & timeliness_{BMP280}\left[low\right]\;\vee \;accuracy_{LSM6DSL}\left[low\right], \end{array}$$

(17) $$\begin{array}{cc} R_{2}:Data\;quality\left[poor\right]= & consistency_{temperature}\left[high\right]\;\vee \;timeliness_{LSM6DSL}\left[high\right]\;\vee \\ & timeliness_{TMP461}\left[high\right]\;\vee \;timeliness_{BMP280}\left[low\right]\;\vee \\ & accuracy_{LSM6DSL}\left[high\right], \end{array}$$

(18) $$\begin{array}{cc} R_{3}:Data\;quality\left[average\right]= & completeness_{LSM6DSL}\left[high\right]\;\vee \;completeness_{TMP461}\left[high\right]\;\vee \\ & completeness_{BMP280}\left[high\right], \end{array}$$

(19) $$\begin{array}{cc} R_{4}:Data\;quality\left[good\right]= & consistency_{temperature}\left[medium\right]\;\wedge \;completeness_{LSM6DSL}\left[medium\right]\;\wedge \\ & completeness_{TMP461}\left[medium\right]\;\wedge \;completeness_{BMP280}\left[medium\right]\;\wedge \\ & timeliness_{LSM6DSL}\left[medium\right]\;\wedge \;timeliness_{TMP461}\left[medium\right]\;\wedge \\ & timeliness_{BMP280}\left[low\right]\left[medium\right]\;\wedge \;accuracy_{LSM6DSL}\left[medium\right]. \end{array}$$

Equation (16) defines the set of rules for low data quality, given observations with low membership. Rule 2 defines poor data quality readings for sensor values that are too high. The third rule defines the average quality of the data if the completeness is high, which means that more samples were acquired than needed. The last rule specifies the conditions for high data quality if all observations are in the medium range.

### 3.7. Intel Lab Data

**Figure 7:**
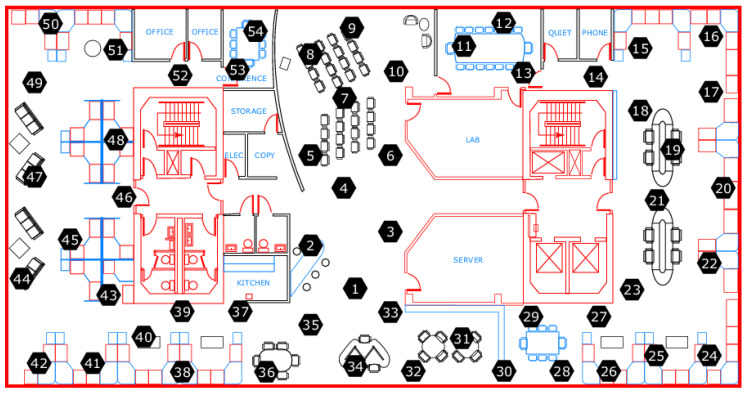
Distribution of 54 sensors in Intel Berkeley Research lab [30].

This data set was collected by Madden et al. [30] in 2004. The authors deployed 54 Mica2Dot wireless sensors equipped with weather boards to measure environmental parameters such as humidity, temperature, light, and sensor voltage at an interval of 31 s. According to the authors, there is a strong correlation between voltage and temperature variation [30]. Additionally, the data sets contain aggregated connectivity data and the x and y positions of the individual sensors in the lab. The spatial distribution of the sensors within the Intel Berkeley Research Lab is shown in Figure 7. The data sets were processed by extracting measurement points from the individual sensors, called motes. Timestamps were processed per mote and sorted in ascending order.

**Figure 8:**
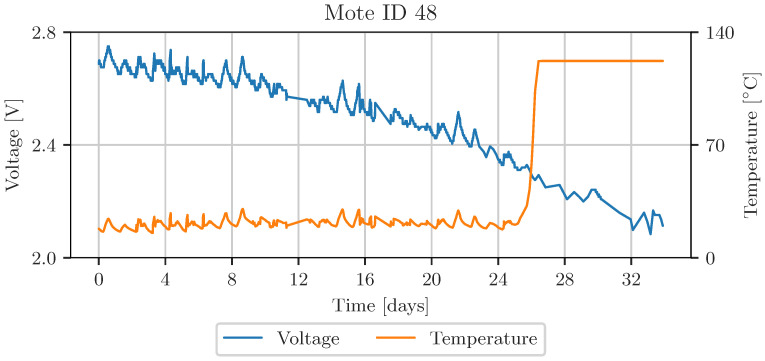
Supply voltage dependency of temperature measurement from sensor mote ID 48.

An exploratory data analysis showed that some motes did not record data over the entire time period, while others showed significant gaps. Motes 46 to 48 were found to have complete recordings and also show the effect of voltage drop in the battery. All three sensors are located close to each other, in the left part of the laboratory, according to Figure 7. Measurements of temperature and voltage acquired by mote 48 are shown in Figure 8. This plot also shows the correlation between temperature and voltage measurement. The accuracy attribute of the Intel Lab Data is calculated from the voltage level. In addition, completeness and timeliness are calculated. Consistency was not computed for this data set because the motes do not carry redundant sensors.

The fuzzy logic rules, derived from the Intel Lab Data, are given in Equations (20)–(22):

(20) $$\begin{array}{cc} R_{1}:Data\;quality\left[poor\right]= & completeness[low]\;\vee \;timeliness[low]\;\vee \\ & timeliness\left[high\right]\;\vee \;voltage_{inv}\left[high\right], \end{array}$$

(21) $$\begin{array}{cc} R_{2}:Data\;quality\left[average\right]= & completeness[high], \end{array}$$

(22) $$\begin{array}{cc} R_{3}:Data\;quality\left[good\right]= & completeness[medium]\;\wedge \;timeliness[medium]\;\wedge \\ & (voltage_{inv}\left[low\right]\;\vee \;voltage_{inv}\left[medium\right]). \end{array}$$

Three rules were derived, representing the possible data quality levels poor, average, and high. The quality attribute of the voltage data was calculated based on the inverse of the actual voltage. This was done by changing the significance of the values, making large values small and small values large. High data quality is assumed when the inverse of the voltage is low or medium. Inverting is helpful because large voltages represent a higher data quality. This way, the accuracy attribute calculated through voltage can be better mapped in the memberships of low, average, and poor. This procedure was chosen to map the characteristics of the data set to the proposed semi-automatic rule definition approach.

## 4. Results

The maximum likelihood and fuzzy logic-based data fusion methods introduced before were applied to the Accelerometer Data Set and the Intel Lab Data. The semi-automatic rule definition was applied to create the fusion algorithms for the used data sets. The behavior of the fusion algorithms is verified by calculating correlation coefficients and evaluating the similarity of the signals.

### 4.1. Accelerometer Data Set

**Figure 9:**
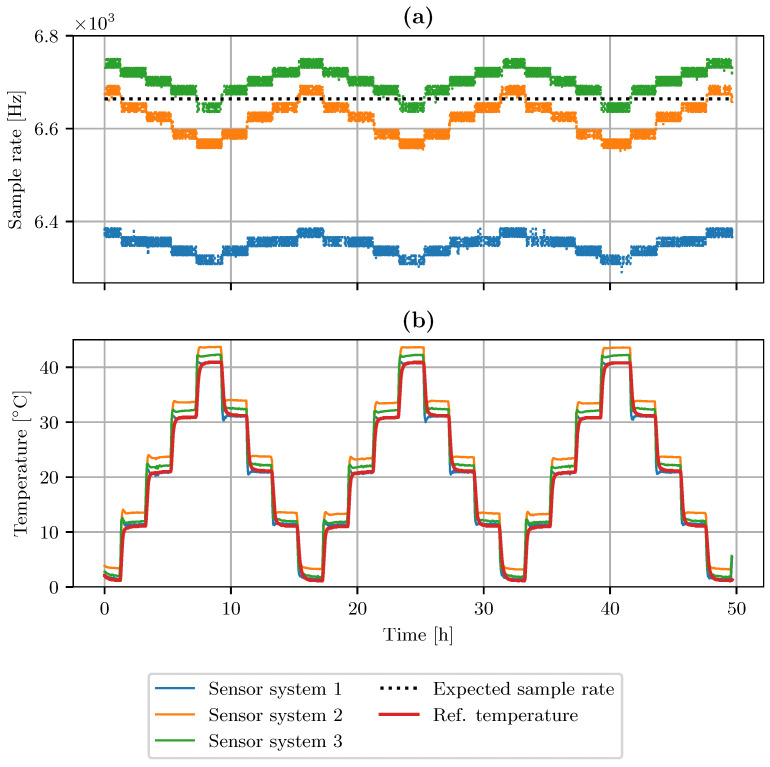
(**a**) Measured and expected sample rate of three LSM6DSL sensor systems. (**b**) Temperature profile of VT 7010 as measured by BMP280 temperature sensors.

Figure 9a shows the measured sample rates of the LSM6DSL accelerometer sensors. Additionally, the black dotted line represents the expected sample rate. The temperature curve in Figure 9b, measured by the BMP280 sensor, also includes the reference temperature curve. The plots show the data points of all three sensor systems. For sensor system 1, we observed a minimum sampling rate of 6283 Hz, a maximum sampling rate of 6385 Hz, and a standard deviation (std) of 22 Hz. System 2 showed a range between 6551 Hz and 6692 Hz, while the standard error was 36 Hz. Sensor system 3 ranges between 6629 Hz and 6749 Hz, with a standard deviation of 26 Hz. The expected sample rate, specified by the data sheet, is highlighted as a black dotted line in Figure 9a. System 2 and 3 meet the specified frequency at 0 °C and 40 °C, respectively.

**Figure 10:**
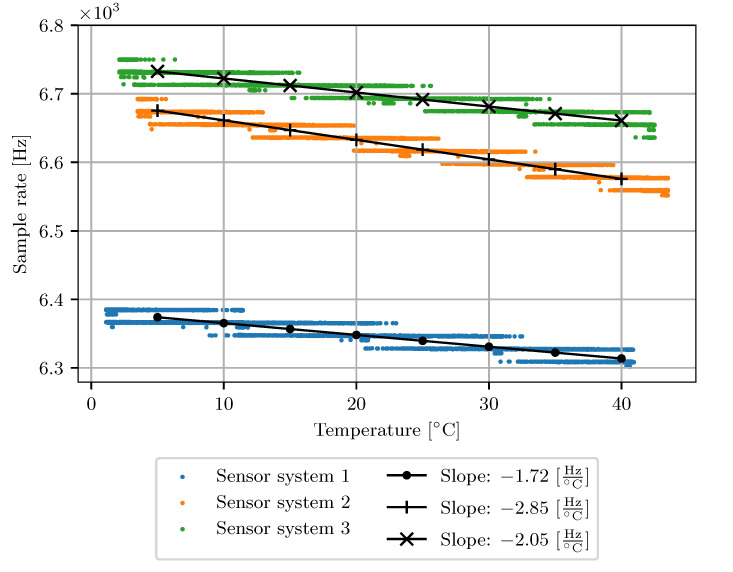
Temperature dependency of LSM6DSL inertial measurement units of three different devices. Slopes are given in Hz/°C.

Figure 10 shows the slopes of the temperature dependency. The black lines represent fitted linear models displaying the temperature coefficient in Hz/°. All three systems show a negative temperature coefficient between −1.72 Hz/°C and −2.85 Hz/°C. The curves show that the ambient temperature influences the sample rate. However, there is a difference between individual devices in the offset and slope of temperature dependence.

Based on the conducted measurement and the acquired data, a total of eight data quality attributes were derived. Table 3 shows a summary of the sensors, the measured values, and the defined quality attributes. The table also includes a summary of the properties of fuzzy logic fusion and MLE fusion, derived by the semi-automatic definition method introduced in Section 3.5 as well as the calculated MSE values of the best fit.

The semi-automatic definition method was applied to all attributes except LSM6DSL sample rate accuracy, using a knowledge-based approach for this attribute. Based on the proposed method, all automatically defined rules consist of normally distributed values. The timeliness is the average time difference between subsequent samples in seconds. A medium timeliness for LSM6DSL of 0.052 s, 0.263 s for TMP461, and 1.052 s for BMP280 is found. Similarly, completeness attributes were calculated for a time slot of one second; thus, the resulting values have a unit of samples per second. The semi-automatic rule definition yields an average completeness of 19.85 S/s for LSM6DSL, 3.98 S/s for TMP461, and 0.99 S/s for BMP280. The completeness of BMP280 was calculated over a time window of 10 s and then normalized to 1 s. For the other values, an interval of 1 s was chosen. The average consistency between the temperature measurements was 0.15 °C. The mean squared error values, used to evaluate the goodness of fit range between 0.1 and 0.05.

**Figure 11:**
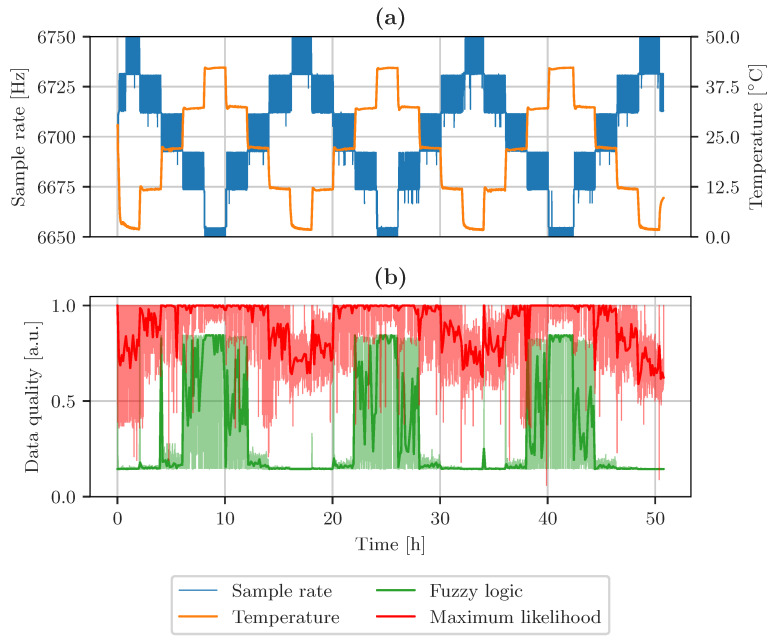
(**a**) Sample rate accuracy of sensor system 3 with changing temperature. (**b**) Data quality indicators based on fuzzy logic and maximum likelihood estimation.

The LSM6DSL sample rate and temperature are plotted in Figure 11a. The graph in Figure 11b shows the fuzzy logic-based data quality indicator as a green line and based on maximum likelihood estimation as a red line. The transparent parts of each graph show the range, while the solid lines show the filtered data quality indicators to illustrate the overall trend of the signal. The data quality level changes with the accuracy of the sample rate. The accuracy is subject to change with the measured temperature. If the sample rate deviates from the expected value, the overall quality of the data is lower. Events of lower data quality are observed at time points 0 h, 16 h, 34 h, and 50 h when using MLE as a data fusion method. High data quality was measured at time points 9 h, 25 h, 41 h. Similarly to the results obtained using MLE, fuzzy logic reports a higher overall data quality if the sample rate is closer to the expected value. Time points 10 h, 25 h, and 40 h represent a high quality of the data indicated by fuzzy logic. Low data quality was indicated at time points around 0 h, 17 h, 34 h, and 50 h. A complete graph with all calculated data quality attributes and both data quality indicators is included in Appendix A.1.

Table 4 shows the Pearson’s correlation coefficients of the data quality attributes used as defined by Table 3 and the aggregated data quality indicators. Strong negative correlations are measured between the fusion approaches and the consistency attribute of the temperature, as well as sample rate accuracy. A negative correlation implies that a lower consistency results in a higher data quality. Based on Figure 11, the negative correlation between the LSM6DSL accuracy attribute and the data quality indicators is interpreted: For this data set, an increase in the sampling rate meant a decrease in data quality, resulting in an overall negative correlation. The timeliness attributes show only a slightly negative correlation, resulting from the deterministic sampling intervals, additionally reflected in the low stand deviations given in Table 3. Positive correlations are also observed between the completeness attributes and the aggregated data quality indicators. Furthermore, a positive correlation of 0.459 between fuzzy logic and likelihood-based data fusion indicates that both tend to increase or decrease for similar inputs. The correlation analysis shows that individual data quality attributes are still present in the aggregated indicators.

### 4.2. Intel Lab Data

The results in this section were acquired using data from sensor Mote IDs 46, 47, and 48. The timeliness and completeness attributes were calculated based on the timestamps provided by the data set, using a time window of 10 min for completeness. One instance of measured values is bound to one timestamp, so specific attributes for a particular measurement cannot be calculated. Furthermore, the voltage level is the quality attribute related to accuracy, as it significantly affects the validity of temperature measurements [30]. Based on the semi-automatic rule definition, the parameters of the membership functions and distributions were calculated. Table 5 shows the parameters of the used fusion algorithms and the mean squared error of the best fit. The best approximation for voltage follows an exponential distribution with a location of 0.39/V and a scale parameter of 0.065/V. The values are given in 1/V because inverted values were used. The location parameter corresponds to 2.56 V. The average timeliness between the samples is 41.5 s. Completeness in the time interval of 10 min was 17 samples. The MSE of the best fitting distributions range between 0.1 and 0.05.

**Figure 12:**
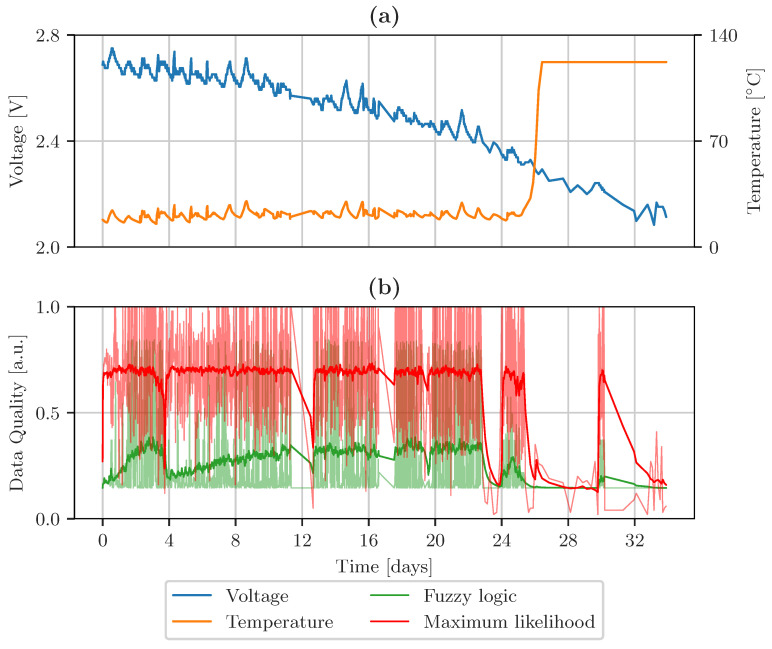
(**a**) Influence of supply voltage on the accuracy of the temperature measurement of Intel Lab mote ID 48. (**b**) Data quality indicators based on fuzzy logic and maximum likelihood estimation.

Figure 12 shows the temperature and voltage acquired by mote 48 in the upper row. The bottom row displays the aggregated data quality levels, calculated by maximum likelihood in red, and fuzzy logic-based data fusion in green. The solid lines in Figure 12b are the averaged data quality indicators, representing the general trend of the signals. The transparent parts of this graph represent the overall quality of the data. The data quality level of both fusion algorithms decreases when the voltage drops and the temperature measurement is corrupted, approximately after 26 days. An additional downtime, after 12 days, leads to worse overall data quality.

In Table 6, the correlation coefficients between the calculated aggregated data quality indicators and the used data quality attributes are shown. We observe positive correlations between voltage, completeness, and the data quality indicators, meaning that higher values of these attributes result in a higher quality of the data, which is in line with our assumptions. A negative correlation between temperature and the indicators represents the fact that a higher temperature corresponds to a lower quality of the data. Lastly, a significant positive correlation of 0.663 between the fused data quality indicators shows that both algorithms report a lower or higher quality of the data for similar inputs. Complete graphs of the used data quality attributes and the calculated data quality indicators are given in Appendix A.2.

## 5. Discussion

This work proposes a framework to aggregate data quality attributes into a single meaningful data quality indicator. The results are discussed in the following section. The Accelerometer Data Set reflects our initial assumption that the temperature of the sensor system influences the overall quality of the data. Based on the proposed knowledge-driven and data-driven approach, a set of rules has been established to aggregate data quality attributes into a single meaningful indicator. Interpretability is gained through the outputs of the indicators themselves. Both indicators, obtained by fuzzy logic and the maximum likelihood method, are in the range between 0 and 1, where higher values indicate higher data quality. The general information content of the fused outputs regarding their inputs was analyzed with a correlation analysis.

Despite the different characteristics of the two used data sets, our approach provided consistent results. In both data sets, the proposed timeliness attributes do not contribute significantly to the overall data quality indicator, leaving room for further improvement. A possible reason for this could be the used metric. The interpretation of the results of the fuzzy logic and maximum likelihood-based approaches shows the same trend of reporting worse data quality for similar inputs. However, fuzzy logic is more responsive to issues related to data quality. This is because the fuzzy rule set can be finely tuned to the desired data quality level. In contrast, the MLE-based approach is completely determined by the distribution used. Tuning this method is only possible by modifying these distributions, making it less flexible but also less effort to construct compared to the fuzzy logic-based approach. This comes at the cost of a less sensitive data quality output. The nature of MLE is to compute the likelihood given a set of observations; if, for example, only one input value is of low quality, statistically, the overall data quality could still be high. This work covers only a limited number of membership functions and distributions. The supported algorithmic properties could be extended further to give developers more degrees of freedom when designing a quality fusion system. In addition, the algorithms have not been implemented directly in the sensor systems. Furthermore, the noise level of the fused data quality indicators requires further analysis. A possible solution could be to apply a moving average filter, limiting the dynamics of the data quality indicator. Additionally, to address the real time requirements as well, a more sophisticated approach can be considered, such as variable mode decomposition [20]. Moreover, a combined approach using compensation for the sampling rate deviation together with the proposed fusion framework could be used to enhance the sensor output with further information.

Compared to previous work in the field of data quality indication in sensor systems, our framework provides an end-to-end approach to building a quality fusion algorithm. Starting with data and domain knowledge, statistical properties, and fuzzy rule sets are extracted. Based on these, the actual data-fusion algorithms are parameterized. Wu et al. [14] proposed a Q-Fusion approach based on normalized data quality attributes. Our approach, in contrast, allows one to directly input the quality attributes into the fusion algorithm, giving better traceability and reducing the overall complexity of the data processing. The work of D’Aniello et al. [6] and Ehikioya et al. [7] proposed using fuzzy logic to solve data quality-related use cases. D’Aniello et al. limit their work to virtual sensors with service level agreements. Ehikioya et al. proposed the use of fuzzy methods for data quality-related issues in business and enterprise data. Our approach, in contrast, can be directly implemented on the sensor itself, working on raw sensor data. Furthermore, we extend existing methods by the semi-automatic membership definition, capable of incorporating domain knowledge. Two fusion algorithms are part of the framework, giving users an alternative between the more complex and customizable fuzzy logic or the more intuitive but less sensitive MLE-based approach. Further research may include the extension of this framework to distributed sensors. In this scenario, especially the timeliness attribute needs special consideration, since the transmission medium has significant influence on data transmission times. A possible direction could be to advance the calculation of completeness attributes with spatiotemporal metrics. Another extension could include processing of time series rather than single time steps. A limitation of the purely knowledge-driven approach is the possibility of introducing a bias to higher or lower data quality, depending on the data used to generate the fusion algorithms. Furthermore, information not present in the initial input data is not considered a data quality-related event in the final fusion algorithm.

In the next step, we aim to port the proposed fusion methods to sensor systems to show their feasibility in live scenarios. While porting, the latency of the fusion algorithm, running in a resource-constrained system, has to be addressed. Furthermore, a suitable description of the data quality indicators and the necessary metadata are required.

## Figures and Tables

**Table 1:** Commonly defined data quality attributes. The table is in alphabetical order, sorted by first author name.

Author	Reported Attributes
Bisdikian et al. [16]	Timeliness, accuracy, reliability, throughput, cost
Ehikioya et al. [7]	Precision, timeliness, completeness, correctness, usability
Gutierrez et al. [17]	Accuracy, reliability, spatial precision, completeness, communication reliability, consistency, currency, volatility, timeliness, availability, adequacy
Kuka et al. [18]	Timeliness, accuracy, completeness, consistency, confidence
Vedurmudi et al. [9]	Accuracy, completeness, timeliness, consistency, battery level, calibration data, operating condition, sampling rate

**Table 2:** Number of samples acquired per sensor system and per individual sensor.

Sensor System	LSM6DSL	TMP461	BMP280
1	3,486,952	699,134	174,846
2	3,502,076	702,701	175,631
3	3,501,173	702,112	175,572

**Table 3:** Measurements and data quality attributes for the Accelerometer Data Set. The column MLE explains the properties of the fitted distribution functions and the mean squared error ϵ obtained during the fit. The column fuzzy logic describes the properties of the fuzzy logic membership functions for the specified data quality attributes.

Sensor	Measured Value	Quality Attribute	Unit	MLE	Fuzzy Logic
LSM6DSL	Sample rate	Accuracy	Hz	normal; ϵ = 0.09 μ = 6664, σ = 100	low: a = 6464; b = 6564 medium: μ = 6664; σ = 100; high: c = 6764; d = 6864
LSM6DSL	Sample rate	Timeliness	Second	normal; ϵ = 0.07 μ = 0.052, σ = 0.012	low: a = 0.028; b = 0.04 medium: μ = 0.052; σ = 0.012; high: c = 0.065; d = 0.077
LSM6DSL	Sample rate	Completeness	$\frac{\mathrm{Samples}}{\mathrm{Second}}$	normal; ϵ = 0.1 μ = 19.8, σ = 0.6	low: a = 18.6; b = 19.2 medium: μ = 19.8; σ = 0.6; high: c = 20.5; d = 21.1
TMP461	Temperature	Timeliness	Seconds	normal; ϵ = 0.05 μ = 0.263, σ = 0.031	low: a = 0.2; b = 0.23 medium: μ = 0.263; σ = 0.031; high: c = 0.3; d = 0.33
TMP461	Temperature	Completeness	$\frac{\mathrm{Samples}}{\mathrm{Second}}$	normal; ϵ = 0.1 μ = 3.98, σ = 0.14	low: a = 3.7; b = 3.84 medium: μ = 4.0; σ = 0.14; high: c = 4.12; d = 4.26
BMP280	Temperature	Timeliness	Second	normal; ϵ = 0.05 μ = 1.052, σ = 0.14	low: a = 0.78; b = 0.92 medium: μ = 1.052; σ = 0.14; high: c = 1.19; d = 1.33
BMP280	Temperature	Completeness	$\frac{\mathrm{Samples}}{\mathrm{Second}}$	normal; ϵ = 0.1 μ = 0.99, σ = 0.03	low: a = 0.93; b = 0.96 medium: μ = 0.99; σ = 0.03; high: c = 1.03; d = 1.06
TMP461, BMP280	Temperature	Consistency	°C	normal; ϵ = 0.1 μ = 0.15, σ = 0.065	low: a = 0.01967; b = 0.08465 medium: μ = 0.15; σ = 0.065; high: c = 0.2146; d = 0.2796

**Table 4:** Pearson’s correlation coefficients between data quality indicators and data quality attributes of Accelerometer Data Set.

	Accuracy	Timeliness	Completeness	Timeliness	Completeness	Timeliness	Completeness	Temperature
	LSM6DSL	LSM6DSL	LSM6DSL	TMP461	TMP461	BMP280	BMP280	Consistency
MLE	−0.742993	−0.024866	0.104197	−0.025454	0.040001	−0.029806	0.043472	−0.834314
Fuzzy logic	−0.674421	−0.007066	0.193791	−0.029857	0.011291	−0.032024	0.185742	−0.753731

**Table 5:** Measurements and data quality attributes for the Intel Lab Data set. The column MLE explains the properties of the fitted distribution functions and the mean squared error ϵ obtained during the fit. The column fuzzy logic describes the properties of the fuzzy logic membership functions for the specified data quality attributes.

Sensor	Measured Value	Quality Attribute	Unit	MLE	Fuzzy Logic
Voltage	Battery voltage	Accuracy	$\frac{1}{V}$	exponential; ϵ = 0.07 μ = 0.39, β = 0.026	low: a = 0.33, b = 0.36 medium: μ = 0.385, σ = 0.026 high: a = 0.41, b = 0.44
Voltage	Battery voltage	Timeliness	Seconds	normal; ϵ = 0.05 μ = 41.5, σ = 539.7	low: a = 31.56, b = 36.6 medium: μ = 41.6, σ = 5; high: c = 46.6, d = 51.6
Voltage	Battery voltage	Completeness	$\frac{\mathrm{Samples}}{10\;\min}$	normal; ϵ = 0.1 μ = 17.0, σ = 2.6	low: a = 11.7, b = 14.4 medium: μ = 17.0, σ = 2.65; high: c = 19.7; d = 22.3

**Table 6:** Pearson’s correlation coefficients between data quality indicators, measured values, and data quality attributes of Intel Lab Data.

	Temperature	Voltage	Timeliness	Completeness
MLE	−0.233428	0.182690	−0.049961	0.295445
Fuzzy logic	−0.101262	0.025131	−0.015041	0.152983

**Figure A1:**
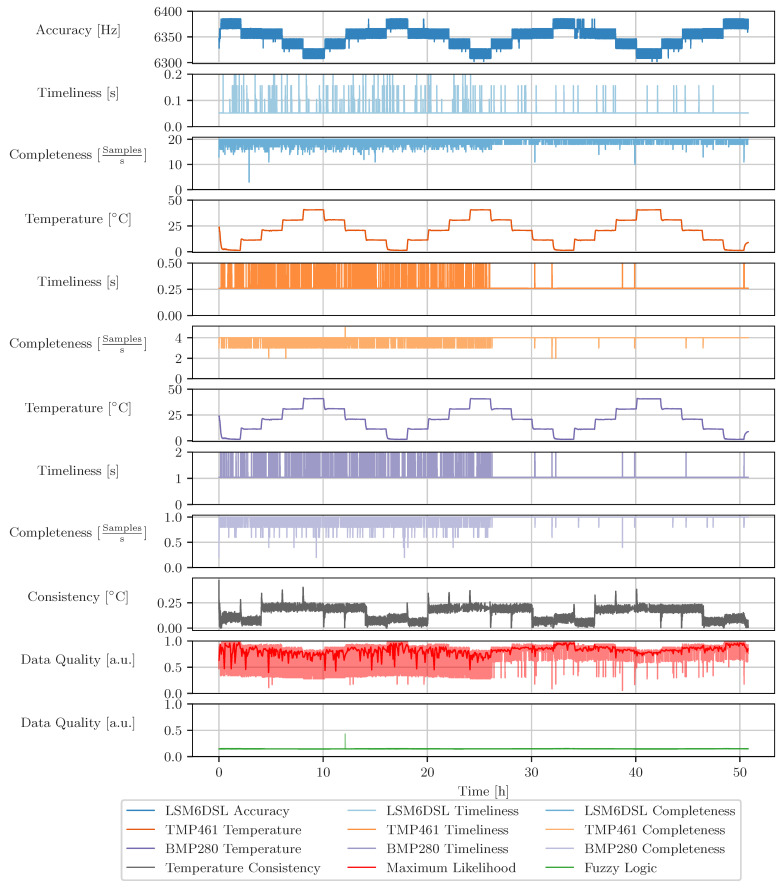
Accelerometer Data Set, sensor system 1, data quality attributes, measured values, and data quality indicators.

**Figure A2:**
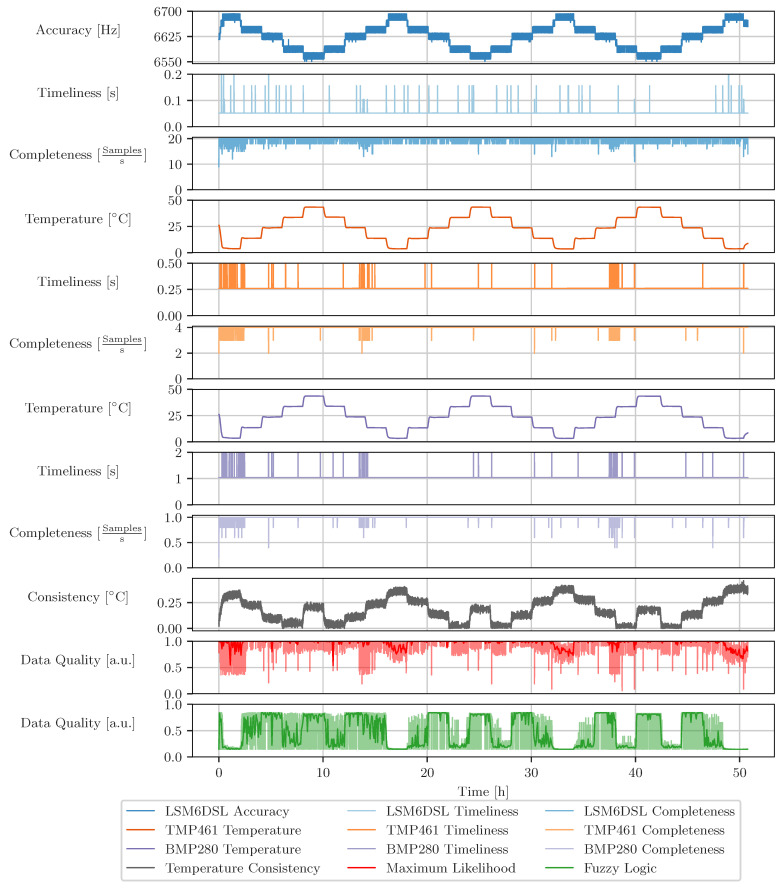
Accelerometer Data Set, sensor system 2, data quality attributes, measured values, and data quality indicators.

**Figure A3:**
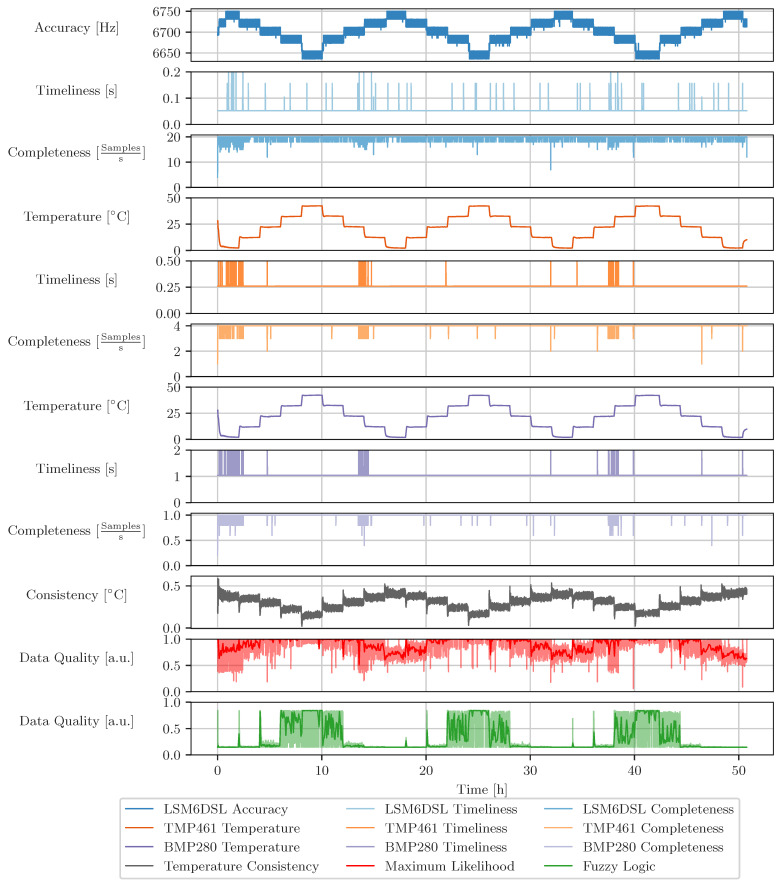
Accelerometer Data Set, sensor system 3, data quality attributes, measured values, and data quality indicators.

**Figure A4:**
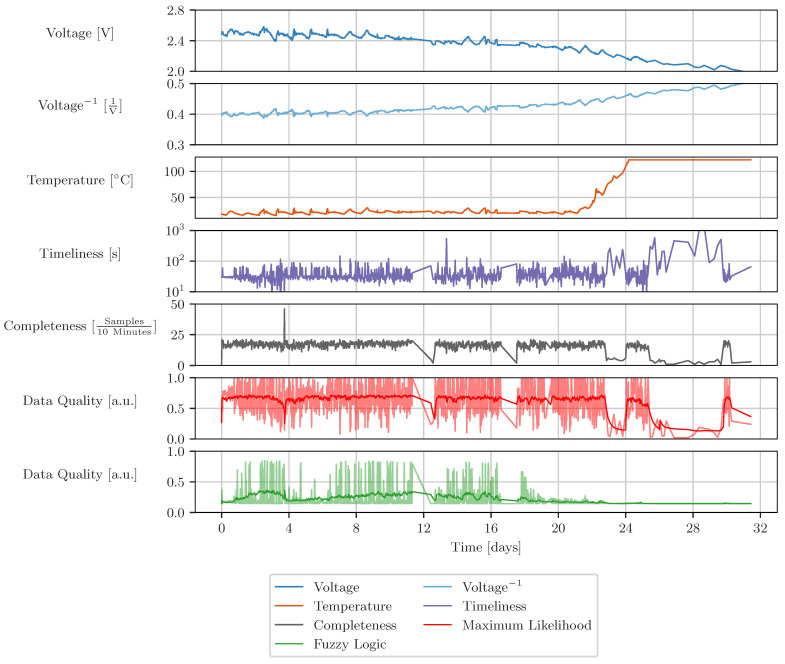
Intel Lab Data Mote ID 46, data quality attributes, measured values, and data quality indicators.

**Figure A5:**
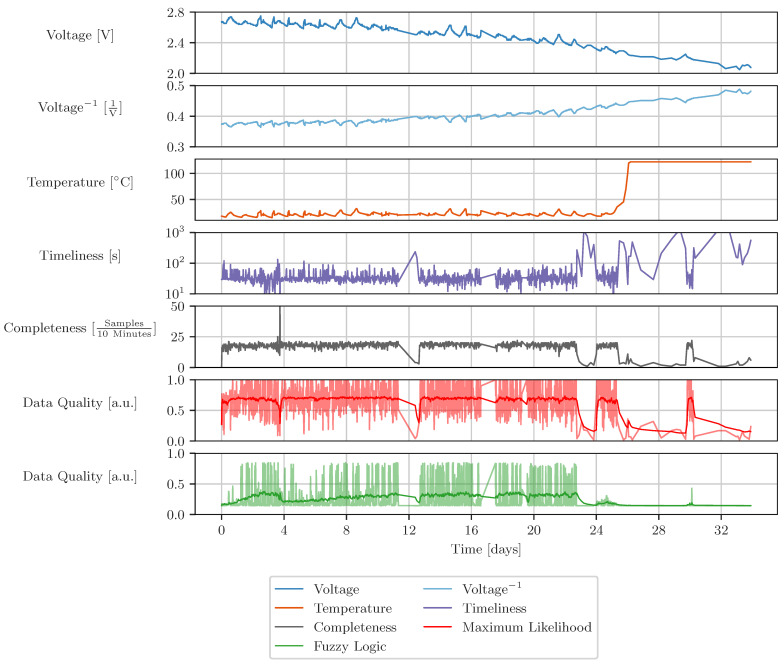
Intel Lab Data Mote ID 47, data quality attributes, measured values, and data quality indicators.

**Figure A6:**
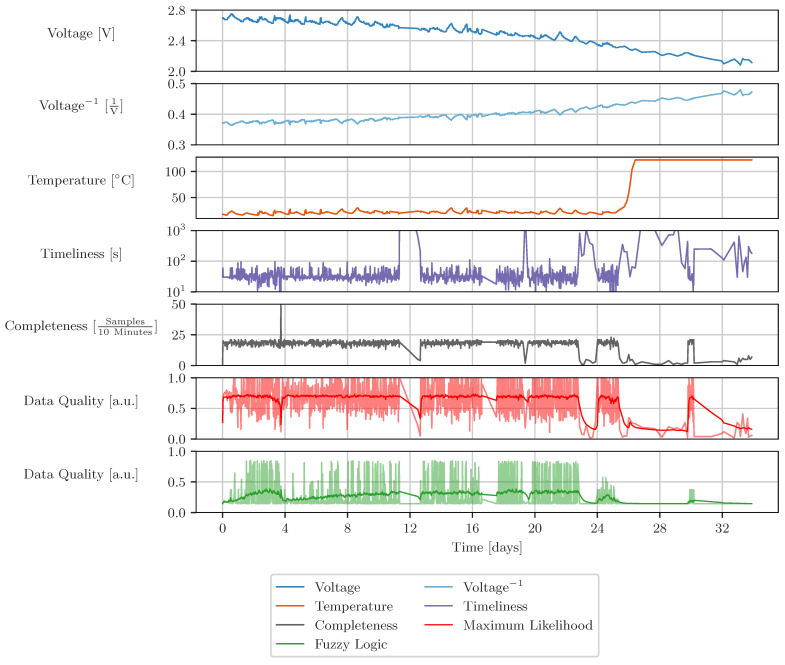
Intel Lab Data Mote ID 48, data quality attributes, measured values, and data quality indicators.

## Data Availability

The data underlying this article will be shared on reasonable request from the corresponding author.

## Appendix A

### Appendix A.1. Accelerometer Data Set

This section provides complete graphs of all data acquired from sensor systems 1 to 3. The graphs in Figure A1, Figure A2 and Figure A3 include the raw measured data points, which are the temperature TMP461 and BMP280, as well as the LSM6DSL sample rate. Additionally, the calculated data quality attributes for each sensor signal as defined in Table 3 are ploted. The last two rows show the quality of the data calculated by fuzzy logic and maximum likelihood estimation. The solid lines in the graphs of the data quality indicators represent the averaged trend of the signals. The slightly transparent parts of the plots are the original signals.

### Appendix A.2. Intel Lab Data

This section provides full graphs of the raw sensor signals from Mote IDs 46, 47, and 48 in Figure A4, Figure A5 and Figure A6. They include the calculated data quality attributes as specified in Table 5, as well as the aggregated data quality indicators. The solid lines in the graphs of the data quality indicators represent the averaged trend of the signals. The slightly transparent parts of the plots are the original signals. The y-scale of the timeliness attribute is in logarithmic scale, due to large deviations of the timeliness values in the data set.
